# Good results with minimally invasive unicompartmental knee resurfacing after 10-year follow-up

**DOI:** 10.1007/s00590-017-2079-5

**Published:** 2017-11-22

**Authors:** Martin H. Redish, Peter Fennema

**Affiliations:** 1 Parkridge Bone and Joint, 2205 McCallie Ave. Suite 102, Chattanooga, TN 37404 USA; 2 AMR Advanced Medical Research GmbH, Hofenstrasse 89b, 8708 Männedorf, Switzerland

**Keywords:** Osteoarthritis, knee, Arthroplasty, replacement, knee, Unicompartmental knee replacement, Cementation, Survival analysis

## Abstract

The current study was designed to determine (1) 10-year implant survival and (2) patient’s self-reported functional outcome in a single surgeon’s consecutive cohort of patients who had undergone minimally invasive unicondylar resurfacing with a modified cementation technique utilizing a cobalt–chromium femur/inlaid all-PE tibia, fixed-bearing unicompartmental prosthesis. We included 344 consecutive patients (361 knees) who had received the study device between January 2002 and December 2005 in this retrospective study. After 10 years, 78 patients (78 knees) had died, 59 (59 knees) were lost to follow-up and four (four knees) did not participate. Thirteen knees (11 patients) were revised after a mean of 5.8 ± 1.9 years. Hence, the study population at follow-up comprised 192 patients (207 knees). Ten-year implant survival was 94.6% (95% confidence interval, 90.9–96.8%). The Forgotten Joint Score and Oxford Knee Score were 68.9 ± 28.9 and 39 ± 9.1, respectively. Excellent survivorship and clinical outcomes were obtained with UKA with an inlaid all-PE tibia with a modified cementation technique.

## Introduction

Total knee arthroplasty (TKA) achieves excellent outcomes on a range of measures. However, patients may experience significant limitations, such as impaired functional activity [1], and residual pain [2]. Unicompartmental knee arthroplasty (UKA) is a less-invasive procedure, resulting in faster rehabilitation, greater preservation of bone stock, reduced blood loss, and a lower risk of infection in comparison with TKA [3–7]. Furthermore, knee kinematics following UKA has been reported to be closer to those of the native knee than with TKA [8–10]. As such, UKA may represent a valuable alternative to TKA for patients who do not need the more invasive procedure [11]. Nevertheless, 50% of conversions from UKA to TKA have significant bone defects, and stemmed implants and/or augments are required in up to 80% of cases [12–15].

The Repicci II (Biomet, Inc, Warsaw, IN, USA) is a cobalt–chromium femur/inlaid all-polyethylene tibia, fixed-bearing unicompartmental prosthesis that is used with a minimally invasive surgical (MIS) technique. In contrast to the commonly used resection UKA, this resurfacing design maintains all the bone stock required for conversion to primary TKA [16]. The popularity of the Repicci prosthesis has waned recently due to variable survivorship outcomes, the need for a freehand bone sculpting, and fears over subsidence of the tibial inlay. While good short- and mid-term functional results have been reported [17, 18], and a study by the device inventor reported low revision rates after 8 years [19], one investigation [18] reported lower mid-term survivorship and rates of revision than those recorded for other UKA systems [20–22]. However, the longevity of UKA is highly dependent on the surgical technique, as well as on implant position and alignment [23, 24].

The study device was adopted in our clinic with a modified cementation technique. We aimed to assess whether this procedure is successful in our heterogeneous patient population. In patients under 60, the less a UKA compromises any future revisions is important. If avoiding revision TKA is thought of as an endpoint to avoid, if successful, this technique when utilized in the algorithm of care, may put off primary TKA for a significant amount of time. In patients over 75, if this procedure is safe, it may prevent elderly patients from the arduous recovery of TKA.

## Materials and methods

We retrospectively recruited consecutive patients with isolated medial or lateral osteoarthritis who underwent cemented unicondylar resurfacing with the Repicci II UKA system between January 2002 and December 2005.

All patients who had received the Repicci II UKA system implant from the first author were eligible. Patients in the study all had Kellgren–Lawrence Grade 4 changes on the affected side with no more than maximum 2 mm joint space on weight-bearing anteroposterior radiographs. Medical comorbidities were across the spectrum, with this procedure often favoured over TKA in the presence of more severe medical conditions due to its safety [25]. Activity levels of patients factored into choice of UKA versus TKA, with UKA favoured for those wanting more active lifestyles (such as hiking and farming). Patients ineligible for resurfacing UKA were those who had inflammatory diseases or significant involvement of the contralateral compartment, as detected at intraoperative arthroscopic examination. Patients with isolated lateral cartilage lesions, particularly those adjacent to the femoral notch, were included; patients with diffuse weight-bearing lesions or with symptomatic patellofemoral osteoarthritis underwent TKA. The decision is also affected by the patient’s age, weight and life expectancy, as well as their personal preference.

Patients were recruited consecutively from the institution’s surgery list, beginning with those operated on first. To minimize attrition, at least three attempts were made to contact each patient, with those who did not respond to a telephone call contacted at least twice by mail. In addition, the patient’s general practitioner was contacted, if known. The patients were informed by the investigator as to the purpose of the study, and invited to participate. The records of patients who consented to take part in the study were reviewed, and patient variables, including baseline age, sex and body mass index (BMI), were extracted. The Forgotten Joint Score (FJS) [26] and the Oxford Knee Score [27] questionnaires were mailed to the patients. The FJS is a 12-item patient-reported outcome questionnaire specifically designed to identify the awareness of the replaced knee or hip joint during various daily life activities. The score ranges from 0 to 100 [26]. A high score indicates good outcome, i.e. a high degree of “forgetting” the artificial joint. The Oxford Knee Score (OKS) is a 12-item patient-reported outcome questionnaire that was developed to assess function and pain after knee arthroplasty surgery. The score ranges from 0 (worst outcome) to 48 (best outcome) [27]. Each subject was informed of their right to refuse to participate or to withdraw their consent at any time. IRB approval was obtained prior to study commencement.

The primary endpoint was implant survival, with device explantation for any reason as the event of interest. For patients who are alive and unrevised, secondary endpoints were the FJS and the Oxford Knee Score, while those for patients who had undergone revision were the date and reason for revision, and the components that were explanted. For patients who had died, the date of death was recorded. For patients lost to follow-up, the last time-point that the prosthesis was known to be in situ was obtained. Patients who experienced a revision and subsequently died were included as revised patients. All clinical data was verified, and only depersonalized data was recorded.

All patients underwent a modification of the Repicci technique [16]. The standard operating table was turned backwards, with the unaffected leg placed in a stirrup holder and the affected leg left free. For medial knees, an arthroscopic post was placed laterally to allow valgus stress and lifting of the leg to obtain more flexion, if needed. For lateral knees, an arthroscopic leg holder was placed.

**Fig. 1:**
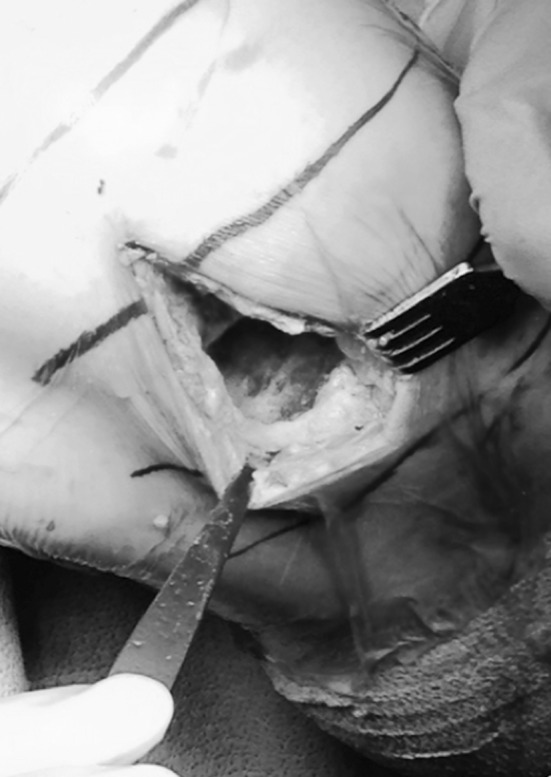
Surgical incision from the superior pole of the patella to just above the tibial tubercle

A brief arthroscopic examination was performed to confirm whether to perform UKA or TKA. If it was UKA, the incision was made from the superior pole of the patella to just above the tibial tubercle (Fig. 1). The capsule was incised in line with the skin incision and extended proximally just into the muscular area. A small portion of the patella edge was resected. The knee was exposed and held in a neutral position at 100° of flexion. Using a saw blade, an 8–12-mm posterior femoral condyle cut was made in line with the posterior shaft. The cut edge was rounded off with a bur and the meniscus removed entirely. For medial replacement, with the knee in flexion and valgus, a small Hohman retractor was placed over the medial edge of the tibial plateau, and the tibial inlay was prepared using round and cylindrical burrs. The inlay was placed as far medial as possible, leaving a very thin rim on the medial side.

**Fig. 2:**
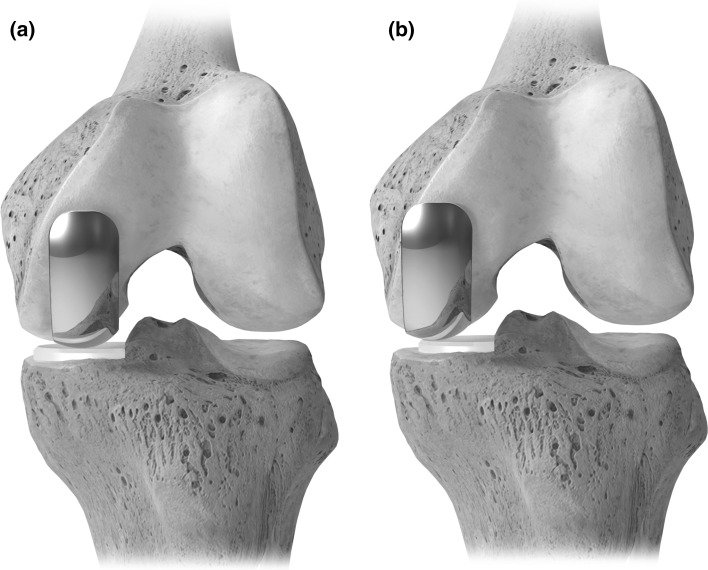
Positioning of the femoral component laterally and close to the femoral notch. Figure 4a shows correct femoral component placement; Fig. 4b shows incorrect positioning

The tibial trial was placed into the defect. The objective was a neutral position (3° varus inclination), with the medial edge sitting 2–4 mm proud, depending on the degree of varus inclination of the patient’s tibial plateau. Femoral preparation was performed freehand, with burrs down to the subchondral bone, keeping the femoral component position laterally, close to the femoral notch (Fig. 2a). The hole was created using a standard cutting jig with a drill, while a small saw and 2-mm wire-passing burr was used for the slot. The trial components were then placed to confirm the proper fit and ensure that the articulating components maintained contact through the full range of motion. Coronal plane limb alignment was checked to avoid overcorrection into valgus. The trials were removed and high-pressure pulse lavage performed, on the tibial inlay preparation. This is done by a malleable frazier suction bent to a right angle at the end and inserted into a standard pulse lavage device. This enables deeper cement penetration into the tibia by more thoroughly cleaning out marrow contents from the subchondral bone [28]. Low viscosity bone cement (CMW 3, DePuy, Warsaw, IN) was introduced into the tibial defect as soon as it became workable to allow for deep penetration. The 6.5–8.5 mm all-polyethylene inlayed tibial component was placed on the sclerotic subchondral bone no deeper than 4–5 mm from the joint surface, with direct firm pressure to fully seat the component. Then, the femoral component was carefully cemented, and femoral osteophytes removed. The tissues were infiltrated with Marcaine plus epinephrine. The knee was then irrigated and closed over a 1/8 in. standard hemovac drain.

For lateral resurfacings, the posterior femoral cut averaged 6 mm in thickness. The tibial component was internally rotated and contact areas with the femur carefully marked throughout flexion and extension. Placement of the femoral component corresponded with these markings.

Patients were ambulated the day of surgery with weight bearing as tolerated, and discharged the following day after removal of the drain. Narcotics were discontinued after 1 week. Aspirin was prescribed as deep vein thrombosis prophylaxis for 1 month postoperatively.

Patient demographics and baseline characteristics, as well as outcome data, were recorded. Kaplan–Meier (KM) analysis was used to determine implant survival, including 95% confidence intervals (CI), with revision of any implant component as the endpoint. Multiple linear regression was also performed to determine the influence of joint covariates on the estimates. The continuous predictor variables age and BMI were categorized into four and five levels, respectively. Patient ages between 55 and 65 years and a BMI between 25 and 30 kg/m^2^ were chosen as the reference levels, as they were the most prevalent in the study cohort. *p* < 0.05 was considered statistically significant. Two-tailed tests were used throughout, with Stata/SE, version 12.1 (Stata Corp, College Station, TX, USA) used for all analyses.

## Results

Three hundred and ninety-three consecutive knees were eligible for UKA. Of those, 32 knees scheduled to undergo UKA were switched to TKA following intraoperative arthroscopic examination, leaving 361 knees (344 patients) for inclusion in the study. The mean age at surgery was 70.5 ± 13.1 years, the mean BMI was 30.9 ± 6.1 kg/m^2^, and 188 patients (54.7%) were female. Three hundred forty knees were treated for medial and 21 for lateral compartment osteoarthritis.

All patients were mobilized immediately following surgery and discharged on postoperative day one. Perioperatively, one superficial infection and one hemarthrosis were noted.

**Fig. 3:**
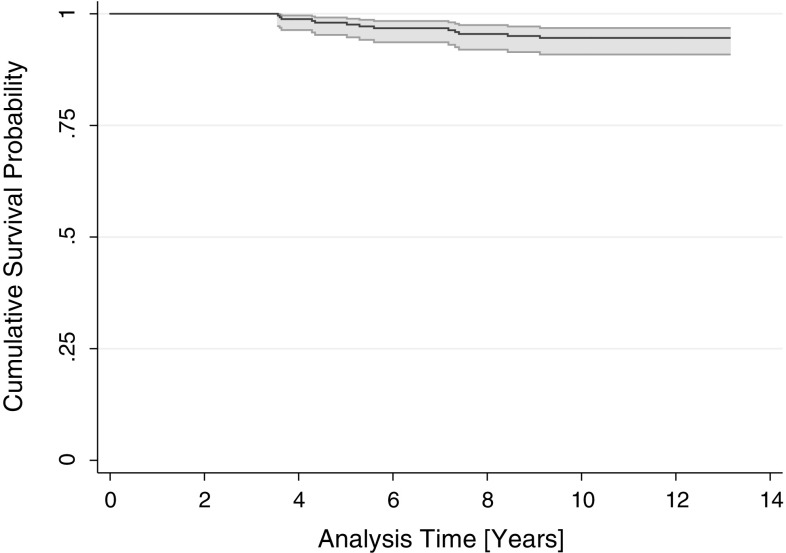
Kaplan–Meier survival plot of revision for any reason

At follow-up, 78 (22.7%) patients (78 knees [21.6%]) had died due to causes unrelated to the procedure and 59 (17.2%) patients (59 [16.3%] knees) were considered lost to follow-up. Four (1.2%) patients (4 [1.1%] knees) did not participate due to being in a hospice or being incapacitated. Eleven (3.2%) patients (13 [3.6%] knees) underwent revision surgery after a mean of 5.8 ± 1.9 years (Table 1). A standard primary TKA with minimum polyethylene insert thickness was used for all revisions. Of the revisions for unexplained medial pain (*n* = 7), five improved after revision, whereas two did not. Ten-year implant survival on Kaplan–Meier analysis was 94.6% (95% CI, 90.9–96.8%) (Fig. 3).

**Table 1:** Overview of revisions with exchange of components

Patient	Gender	Age at surgery (years)	Time of revision (years)	Reason for revision
1	Female	63.7	5.3	Unexplained pain
2	Male	65.4	5.6	Unexplained pain
3	Male	69.7	7.3	Traumatic fracture
4	Female	65.8	7.2	Progression
5	Female	65.6	7.4	Progression
6	Female	60.0	5.0	Femoral loosening
7	Male	58.4	3.6	Tibial component loosening/subsidence
8	Female	59.6	8.4	Unexplained pain
9	Male	64.4	3.6	Gouty arthritis
10	Female	61.6	4.4	Unexplained pain
11	Female	63.4	3.6	Unexplained pain
12	Female	62.7	4.3	Unexplained pain
13	Female	60.9	9.1	Unexplained pain

**Fig. 4:**
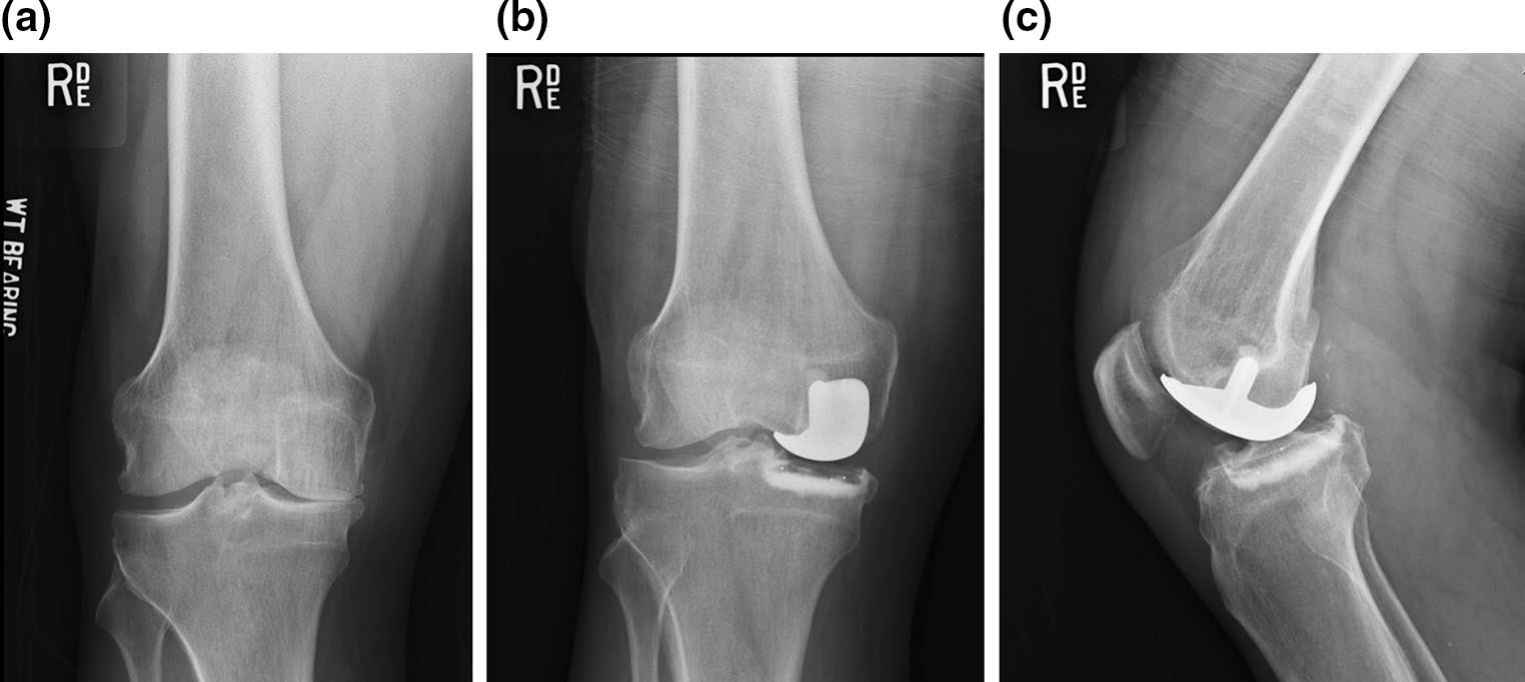
**a** 58-year old male patient with presenting with medial pain. Preoperative anteroposterior radiograph. **b** Postoperative anteroposterior and lateral radiographs. Postoperative radiographs show correct implant position. **c** Lateral radiograph

One hundred and ninety-two patients (207 knees) agreed to follow-up assessment, after a mean follow-up of 10.8 ± 1.1 years. All patients completed the clinical questionnaires. The mean FJS at follow-up was 68.9 ± 28.9, and the mean OKS was 39.5 ± 9.1. Preoperative and postoperative radiographs of a typical patient with a Repicci II knee are shown in Fig. 4.

The results of the multiple linear regression analysis are shown in Table 2. The only statistically significant difference was that patients with a BMI > 40 kg/m^2^ had significantly lower FJS and OKS scores than those with a lower BMI (*p* = 0.009).

**Table 2:** Multiple linear regression analysis

	Forgotten Joint Score			Oxford Knee Score		
	Adjusted mean	95% CI	*p* value	Adjusted mean	95% CI	*p* value
Age (years)						
< 55	60.7	49.1–72.3	0.290	39.0	35.3–42.6	0.293
55–65	66.8 (reference)	56.9–76.7	–	39.8 (reference)	36.7–42.9	–
65–75	69.5	60.3–78.6	0.582	40.4	37.5–43.3	0.968
> 75	76.9	64.4–89.4	0.136	41.6	37.7–45.6	0.003
BMI (kg/m^2^)						
< 5	69.3	55.5– 83.1	0.708	42.1	37.7–46.4	0.293
25–30	66.8 (reference)	56.9–76.7	–	39.8 (reference)	36.7–42.9	–
30–35	67.8	58.5–77.0	0.846	37.8	35.0–40.8	0.223
35–40	72.0	60.1–83.8	0.436	40.0	36.1–43.6	0.968
> 40	48.8	36.0–60.9	0.009	33.3	29.3–37.2	0.003
Male	71.0	61.2–80.8	0.288	40.8	37.7–43.9	0.419

*BMI* body mass index, *CI* confidence interval

## Discussion

Our results demonstrate that good survival and clinical outcomes can be achieved using a resurfacing UKA design with an inlay tibial component, implanted through a minimally invasive surgical technique. In the few UKAs that required conversion to TKA, no stems or augments were needed.

The Australian Orthopaedic Association National Joint Replacement Registry has reported 10-year survival rates of 94.4% for TKA and of 84.9% for UKA in primary OA [29]. The 10-year survival recorded in our series is comparable to those of previous publications. In a retrospective analysis of 136 UKAs using the Repicci system, Romanowski and Repicci [19] reported a revision rate of 7% at 8 years, while Kohan et al. [30] reported 10 year survival of 91.6%. Fuchs et al. [17] described encouraging short-term outcomes with the Repicci system in 379 UKAs at 40 weeks follow-up, with good clinical outcomes and 95% of patients subjectively satisfied with the procedure. In contrast, O’Donnell et al. prospectively followed 100 patients who underwent 114 UKAs with the study system under a short-stay MIS protocol. With 22 patients revised a mean of 6.2 years later, the 9-year survival of the prosthesis was estimated at 78% [18].

Our study design does not allow an understanding of the causes or reasons for the good outcome observed in our study. In the authors’ opinion, there are several potential factors that have contributed to the favorable outcome obtained in our patient series. First, we believe that diagnostic arthroscopy at the time of index surgery is an essential step to reduce the rate of future conversion to TKA due to disease progression in the contralateral compartment. It was decided to perform TKA instead of UKA in ~ 10% of the study population following arthroscopy. Second, the authors feel that the cementation technique employed in this patient series may have contributed to the implant’s longevity.

Third, by resecting 8–12 mm of the posterior femoral condyle, we have allowed enough space to more accurately create the bed for the tibial inlay and removal of excess cement posteriorly. Increasing the flexion gap in UKA has not been shown to be of clinical relevance, possibly due to the cruciate ligaments remaining intact [31]. Braun et al. [32] have shown that resection of the entire posteromedial femoral condyle in athletes to use as bone grafts does not lead to donor site complications, including flexion instability.

Some minor changes to the surgical technique described have been recently implemented. A drain is not deemed necessary any longer [33], tranexamic acid is administered to reduce blood loss [34, 35] and the majority of procedures are performed as outpatients. For medial UKA, none of the patella is resected. In the current series, only one single case of tibial subsidence was observed and therefore no significant association between morbid obesity (BMI > 40) and early loosening was found. Based on the poorer clinical outcome, we nevertheless nowadays are reluctant to use the device in this particular patient population.

In our series, the study system was associated with good clinical outcomes on both the OKS and FJS. OKS compared favorably with 6-month results for TKA from the National Joint Registry for England and Wales [36] and the New Zealand Joint Registry [37]. The FJS in our UKA cohort was significantly higher than that reported in a cohort of TKA patients by Behrend et al. [26], although still lower than their group of healthy controls (Table 3). Our findings coincide with those from Zuiderbaan et al. [38], who found significantly higher FJS values in UKA patients (74.3 ± 24.8) than in TKA patients (59.8 ± 31.5), at a follow-up time of 2 years. Notably, patients with a BMI > 40 kg/m^2^ had lower FJS scores in our series. While our results are highly encouraging, we acknowledge that further studies are required to confirm that they reflect improved outcome after resurfacing UKA. We feel that the FJS is an important method for assessing patient outcome, as the patient’s ability to forget their artificial joint in everyday life could be regarded as the ultimate goal in joint arthroplasty [26]. Our data suggest that resurfacing UKA appears to accomplish this objective better than primary TKA.

**Table 3:** FJS Results compared with results from the literature [26]

	Sex	Mean (SD)
Our values	Male	71.6 (28.5)
	Female	66.6 (29.1)
THA	Male	63.8 (29.2)
	Female	54.7 (32.1)
TKA	Male	56.5 (30.1)
	Female	45.4 (28.0)
Healthy controls	Male	86.6 (17.0)
	Female	79.3 (23.2)

There are several limitations to our study. The study did not include radiographic assessment at follow-up. A relatively large proportion of patients was lost to follow-up. However, the rate of attrition is not uncommonly high for a retrospective study of this length. Another limitation is that all of the procedures were performed by a single surgeon in a single institution. Consequently, the findings are not readily generalizable. In addition, our results were not compared with those for other UKA systems, or with those achieved with TKA. We did not collect baseline data; hence, we were unable to assess the postoperative improvement in OKS scores. We did not document the grade of opposite compartment degenerative disease, and such factors cannot be taken into account when assessing the data. Based on the joint impact of these limitations, inferences from this study should be drawn with caution. Nevertheless, our series represents the largest cohort with long-term follow-up available for this system outside of the inventor’s clinic.

Our long-term follow-up study indicates that, with the described surgical technique, excellent survivorship and clinical outcomes can be obtained with the Repicci II system. Further studies are required to confirm our findings.

## References

[1] Noble PC, Gordon MJ, Weiss JM, Reddix RN, Conditt MA, Mathis KB (2005). Does total knee replacement restore normal knee function?. Clin Orthop Relat Res.

[2] Nashi N, Hong CC, Krishna L (2015). Residual knee pain and functional outcome following total knee arthroplasty in osteoarthritic patients. Knee Surg Sports Traumatol Arthrosc.

[3] Argenson JN, Chevrol-Benkeddache Y, Aubaniac JM (2002). Modern unicompartmental knee arthroplasty with cement: a three to ten-year follow-up study. J Bone Jt Surg Am.

[4] Engh GA (2002). Orthopaedic crossfire—can we justify unicondylar arthroplasty as a temporizing procedure? In the affirmative. J Arthroplasty.

[5] Rougraff BT, Heck DA, Gibson AE (1991). A comparison of tricompartmental and unicompartmental arthroplasty for the treatment of gonarthrosis. Clin Orthop Relat Res.

[6] Knutson K, Lindstrand A, Lidgren L (1986). Survival of knee arthroplasties. A nation-wide multicentre investigation of 8000 cases. J Bone Jt Surg Br.

[7] Iacono F, Raspugli GF, Akkawi I, Bruni D, Filardo G, Budeyri A, Bragonzoni L, Presti ML, Bonanzinga T, Marcacci M (2016). Unicompartmental knee arthroplasty in patients over 75 years: A definitive solution?. Arch Orthop Trauma Surg.

[8] Akizuki S, Mueller JK, Horiuchi H, Matsunaga D, Shibakawa A, Komistek RD (2009). In vivo determination of kinematics for subjects having a Zimmer Unicompartmental High Flex Knee System. J Arthroplasty.

[9] Jung MC, Chung JY, Son KH, Wang H, Hwang J, Kim JJ, Kim JH, Min BH (2014). Difference in knee rotation between total and unicompartmental knee arthroplasties during stair climbing. Knee Surg Sports Traumatol Arthrosc.

[10] Heyse TJ, El-Zayat BF, De Corte R, Chevalier Y, Scheys L, Innocenti B, Fuchs-Winkelmann S, Labey L (2014). UKA closely preserves natural knee kinematics in vitro. Knee Surg Sports Traumatol Arthrosc.

[11] Heyse TJ, Khefacha A, Peersman G, Cartier P (2012). Survivorship of UKA in the middle-aged. Knee.

[12] Wynn Jones H, Chan W, Harrison T, Smith TO, Masonda P, Walton NP (2012). Revision of medial Oxford unicompartmental knee replacement to a total knee replacement: similar to a primary?. Knee.

[13] Chou DT, Swamy GN, Lewis JR, Badhe NP (2012). Revision of failed unicompartmental knee replacement to total knee replacement. Knee.

[14] Barrett WP, Scott RD (1987). Revision of failed unicondylar unicompartmental knee arthroplasty. J Bone Jt Surg Am.

[15] Paredes EB, Sanchez PB, Toledano DS, Gonzalez AIP, Perez SF, de Bobadilla GDF (2017). Total knee arthroplasty after failed unicompartmental knee arthroplasty. Clinical results, radiologic findings, and technical tips. J Arthroplasty.

[16] Repicci J, Hartman JF, Scuderi GR, Tria AJ, Berger RA (2006). Minimally invasive surgery for unicondylar knee arthroplasty: the bone-sparing technique. MIS techniques in orthopedics.

[17] Fuchs S, Strosche H, Tinius W, Gierse H, Gebhardt U (2005). Preliminary remarks on a prospective multicenter study of the Repicci minimally invasive unicondylar knee replacement. Knee Surg Sports Traumatol Arthrosc.

[18] O’Donnell T, Neil MJ (2010). The Repicci II(R) unicondylar knee arthroplasty: 9-year survivorship and function. Clin Orthop Relat Res.

[19] Romanowski MR, Repicci JA (2002). Minimally invasive unicondylar arthroplasty: eight-year follow-up. J Knee Surg.

[20] Vasso M, Del Regno C, Perisano C, D’Amelio A, Corona K, Schiavone Panni A (2015). Unicompartmental knee arthroplasty is effective: ten year results. Int Orthop.

[21] Price AJ, Waite JC, Svard U (2005). Long-term clinical results of the medial Oxford unicompartmental knee arthroplasty. Clin Orthop Relat Res.

[22] Heyse T, Khefacha A, Peersman G, Cartier P (2012). Survivorship of UKA in the middle-aged. Knee.

[23] Chatellard R, Sauleau V, Colmar M, Robert H, Raynaud G, Brilhault J, Societe d’Orthopedie et de Traumatologie de lO (2013). Medial unicompartmental knee arthroplasty: does tibial component position influence clinical outcomes and arthroplasty survival?. Orthop Traumatol Surg Res.

[24] Epinette JA, Brunschweiler B, Mertl P, Mole D, Cazenave A, French Society for H, Knee (2012). Unicompartmental knee arthroplasty modes of failure: wear is not the main reason for failure: a multicentre study of 418 failed knees. Orthop Traumatol Surg Res.

[25] Liddle AD, Judge A, Pandit H, Murray DW (2014). Adverse outcomes after total and unicompartmental knee replacement in 101,330 matched patients: a study of data from the National Joint Registry for England and Wales. Lancet.

[26] Behrend H, Giesinger K, Giesinger JM, Kuster MS (2012). The “forgotten joint” as the ultimate goal in joint arthroplasty: validation of a new patient-reported outcome measure. J Arthroplasty.

[27] Murray D, Fitzpatrick R, Rogers K, Pandit H, Beard D, Carr A, Dawson J (2007). The use of the Oxford hip and knee scores. J Bone Jt Surg Br.

[28] Thompson M, Conditt M, Otto J, Abassi A, Redish M (2010) The importance of a good cement mantle with an all-poly inlay UKA. Poster No. 2121. In: 56th Annual meeting of the Orthopaedic Research Society, New Orleans, LA. Orthopaedic Research Society

[29] AOA (2014) Australian Orthopaedic Association National Joint Replacement Registry, Annual report

[30] Kohan L, Field C, Kerr D (2013). Minimum 10-year follow-up of Repicci unicompartmental knee arthroplasty. Bone Jt J.

[31] Becker R, Mauer C, Starke C, Brosz M, Zantop T, Lohmann CH, Schulze M (2013). Anteroposterior and rotational stability in fixed and mobile bearing unicondylar knee arthroplasty: a cadaveric study using the robotic force sensor system. Knee Surg Sports Traumatol Arthrosc.

[32] Braun S, Minzlaff P, Hollweck R, Wortler K, Imhoff AB (2008). The 5.5-year results of MegaOATS—autologous transfer of the posterior femoral condyle: a case-series study. Arthritis Res Ther.

[33] Zhang Q, Zhang Q, Guo W, Liu Z, Cheng L, Zhu G (2015). No need for use of drainage after minimally invasive unicompartmental knee arthroplasty: a prospective randomized, controlled trial. Arch Orthop Trauma Surg.

[34] Shemshaki H, Nourian SM, Nourian N, Dehghani M, Mokhtari M, Mazoochian F (2015). One step closer to sparing total blood loss and transfusion rate in total knee arthroplasty: a meta-analysis of different methods of tranexamic acid administration. Arch Orthop Trauma Surg.

[35] Hourlier H, Reina N, Fennema P (2015). Single dose intravenous tranexamic acid as effective as continuous infusion in primary total knee arthroplasty: a randomised clinical trial. Arch Orthop Trauma Surg.

[36] Liddle AD, Pandit H, Judge A, Murray DW (2015). Patient-reported outcomes after total and unicompartmental knee arthroplasty: a study of 14 076 matched patients from the National Joint Registry for England and Wales. Bone Jt J.

[37] Pearse AJ, Hooper GJ, Rothwell A, Frampton C (2010). Survival and functional outcome after revision of a unicompartmental to a total knee replacement: the New Zealand National Joint Registry. J Bone Jt Surg Br.

[38] Zuiderbaan HA, van der List JP, Khamaisy S, Nawabi DH, Thein R, Ishmael C, Paul S, Pearle AD (2017). Unicompartmental knee arthroplasty versus total knee arthroplasty: which type of artificial joint do patients forget?. Knee Surg Sports Traumatol Arthrosc.

